# Percutaneous Coronary Intervention-Related Iatrogenic Fistula in Acute Coronary Syndrome

**DOI:** 10.7759/cureus.68348

**Published:** 2024-08-31

**Authors:** Mustafa Yurtdaş, Zeki Dogan

**Affiliations:** 1 Cardiology, Istanbul Atlas University, Faculty of Medicine, Istanbul Medicine Hospital, Istanbul, TUR

**Keywords:** percutaneous coronary intervention complications, management, iatrogenic complication, coronary arterial fistula, acute coronary syndrome

## Abstract

Iatrogenic coronary artery fistula (CAF) can occur following acute myocardial infarction, percutaneous coronary intervention (PCI) procedures, and heart surgery. Iatrogenic CAF linked to PCI has a low incidence rate. Early diagnosis and treatment are crucial when an iatrogenic fistula develops, as it may lead to cardiac tamponade, myocardial infarction, or death. In this report, we present a case of iatrogenic CAF secondary to coronary perforation caused by guidewire-induced trauma, followed by stent implantation and balloon inflation in the context of acute coronary syndrome (ACS). It was successfully managed through prolonged balloon inflation within the previously implanted stent just prior to the rupture zone.

## Introduction

Coronary artery fistula (CAF) is a rare condition connecting a coronary artery to another vessel or cardiac chamber, bypassing the myocardial capillary circulation. Most cases are congenital in origin, affecting approximately 0.002% of the general population. Iatrogenic CAF accounts for a small percentage of all cases [1]. Its incidence ranges from 0.1% to 3%, depending on the treatment approach used in percutaneous coronary interventional (PCI) procedures [1,2]. When iatrogenic CAF occurs, it can quickly lead to cardiac tamponade, myocardial infarction, or death, making prompt treatment essential [1-9]. Herein, we report a case of iatrogenic fistula secondary to coronary artery perforation caused by guidewire-induced trauma and subsequent stent implantation and balloon inflation in the context of the acute coronary syndrome (ACS), which was successfully managed through extended balloon inflation within the previously implanted stent, just before the rupture zone.

## Case presentation

**Figure 1:**
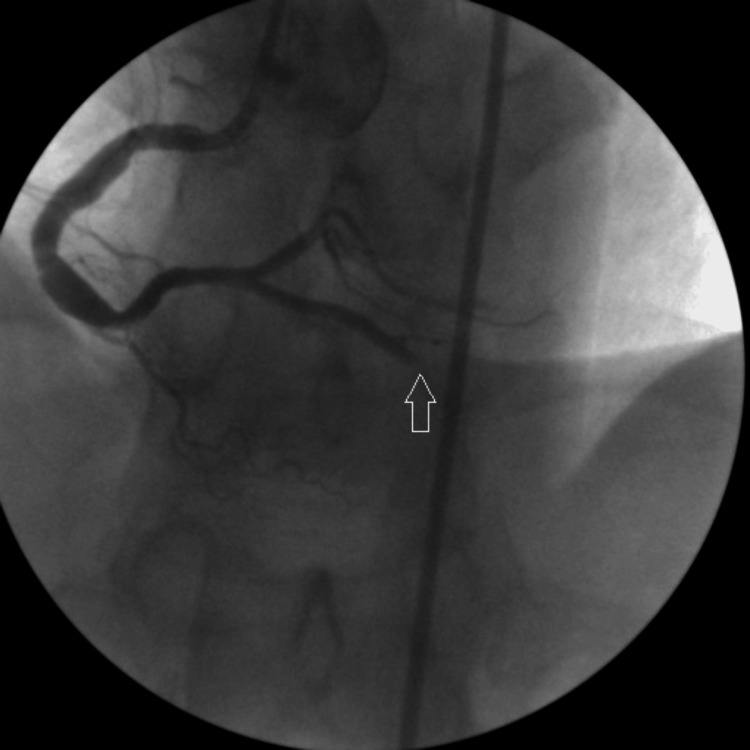
Arrow shows total occlusion of PDA. PDA: Posterior descending artery

A 41‑year‑old male smoker with hyperlipidemia was diagnosed with non‑ST elevation myocardial infarction. After premedication with acetylsalicylic acid (ASA), a loading dose of ticagrelor, and 10,000 IU of unfractionated heparin, the patient underwent coronary angiography (CAG), which revealed total occlusion of the mid-portion of the posterior descending artery (PDA) of the right coronary artery (RCA) (Figure 1).

**Figure 2:**
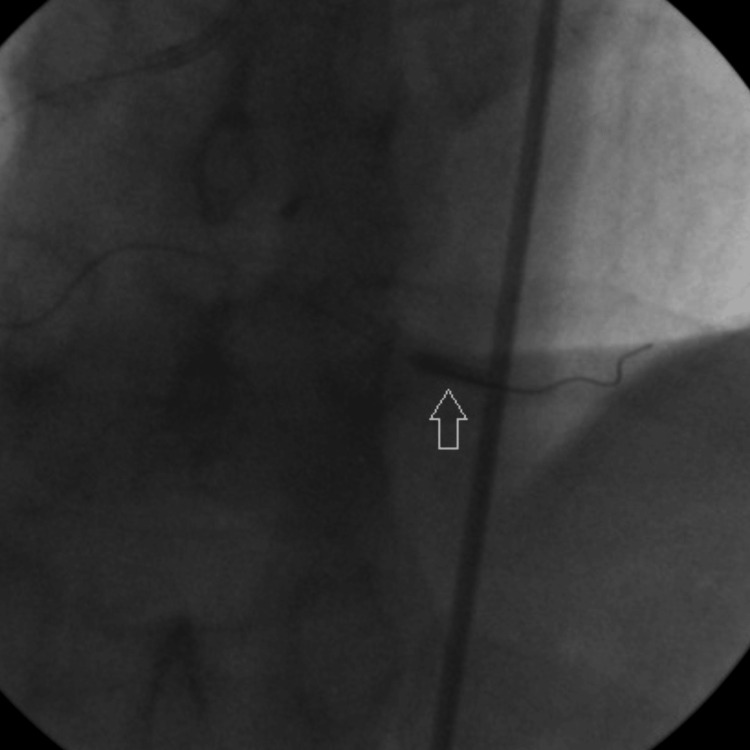
Arrow shows pre-dilation of PDA with 2.5 mm x 9 mm balloon catheter. PDA: Posterior descending artery

**Figure 3:**
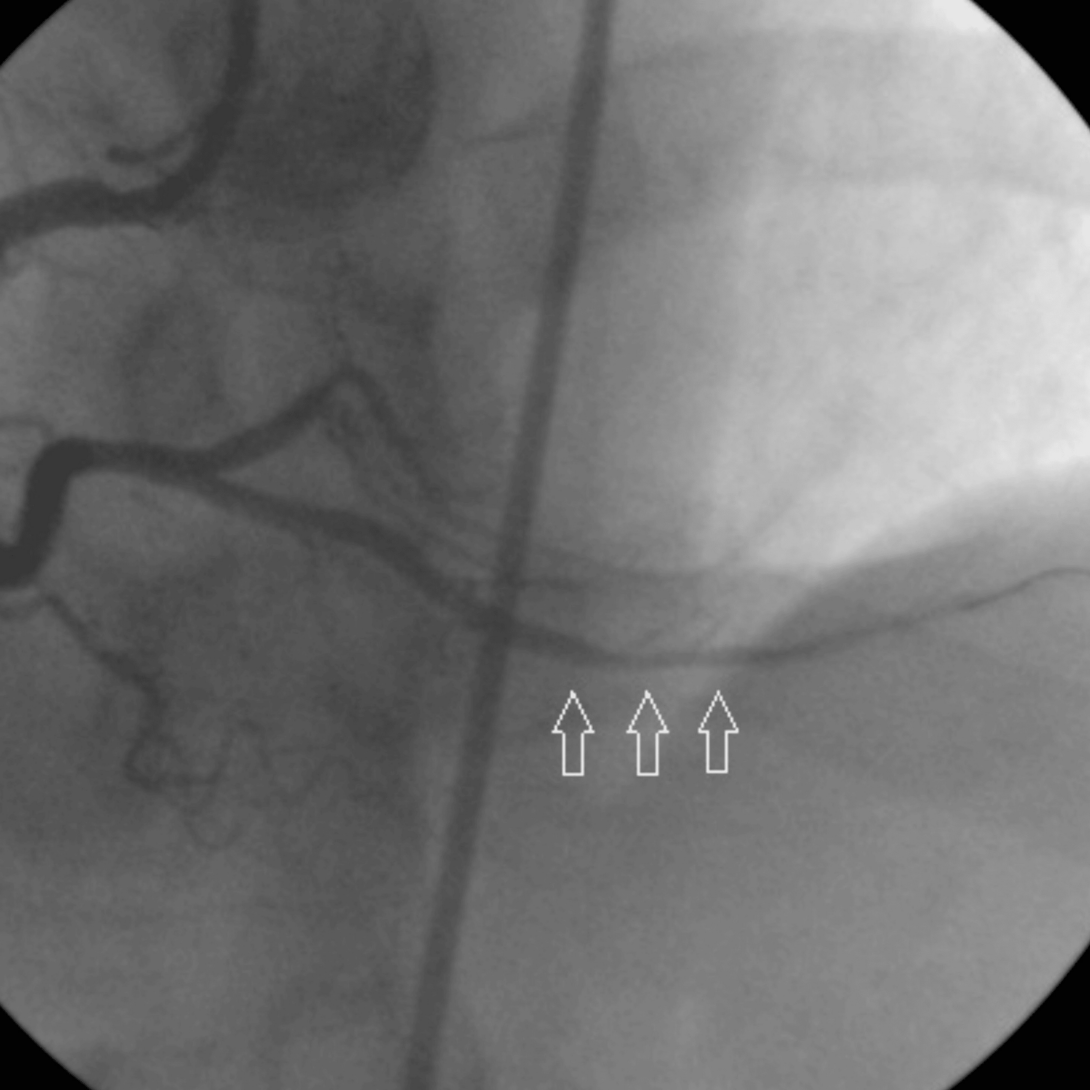
Arrows show patency in PDA. PDA: Posterior descending artery

A 0.014” floppy guidewire was advanced through the occlusion into the distal vessel, culminating in thrombosis with myocardial infarction (TIMI) flow 0. After pre-dilation using a 2.5 mm × 9 mm balloon dilation catheter (Figure 2), the patency in the PDA was restored (Figure 3).

**Figure 4:**
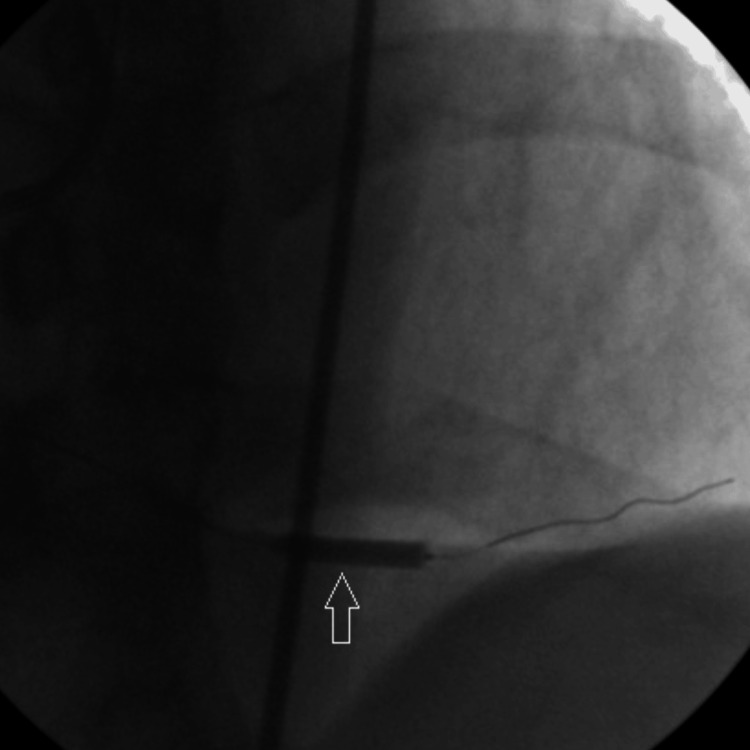
Arrow shows stenting of PDA using drug-eluting stent measuring 3.0 mm x 15 mm. PDA: Posterior descending artery

Thereafter, the culprit lesion was treated with a drug-eluting coronary stent measuring 3.0 mm × 15 mm (Figure 4).

**Figure 5:**
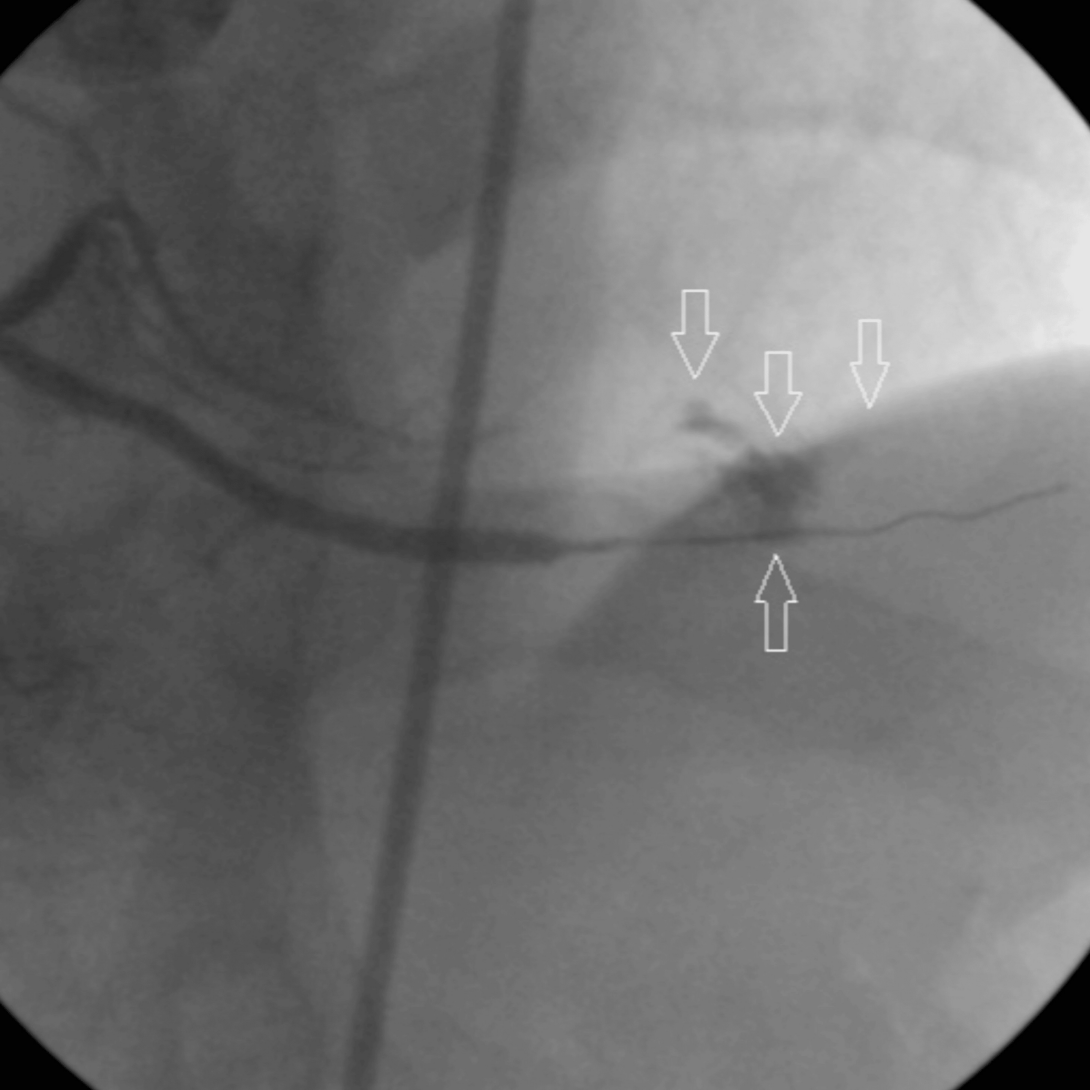
Arrows show fistula after stenting of PDA. PDA: Posterior descending artery

Shortly after, CAG showed a fistula from the PDA to either the left ventricle or the pericardial space (Figure 5).

**Figure 6:**
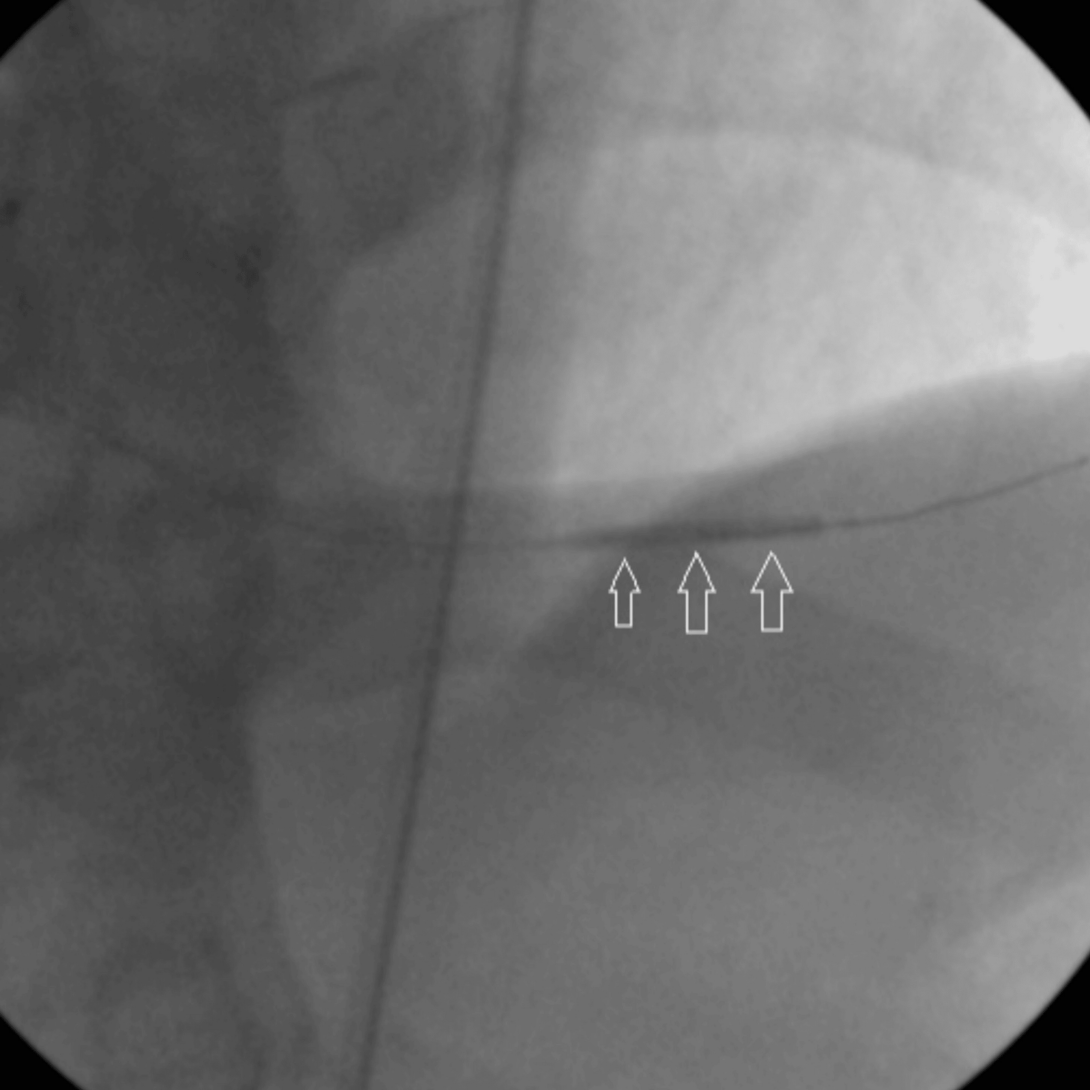
Arrows show prolonged ballooning of ruptured vascular region.

In the initial plan, we quickly conducted the bedtime echocardiography, which revealed no pericardial effusion linked to coronary artery perforation. Subsequently, to seal off the fistula, a new balloon measuring 2.0 mm × 20 mm was advanced into the injured and ruptured vessel, and balloon inflation was carried out for 5 minutes (Figure 6).

**Figure 7:**
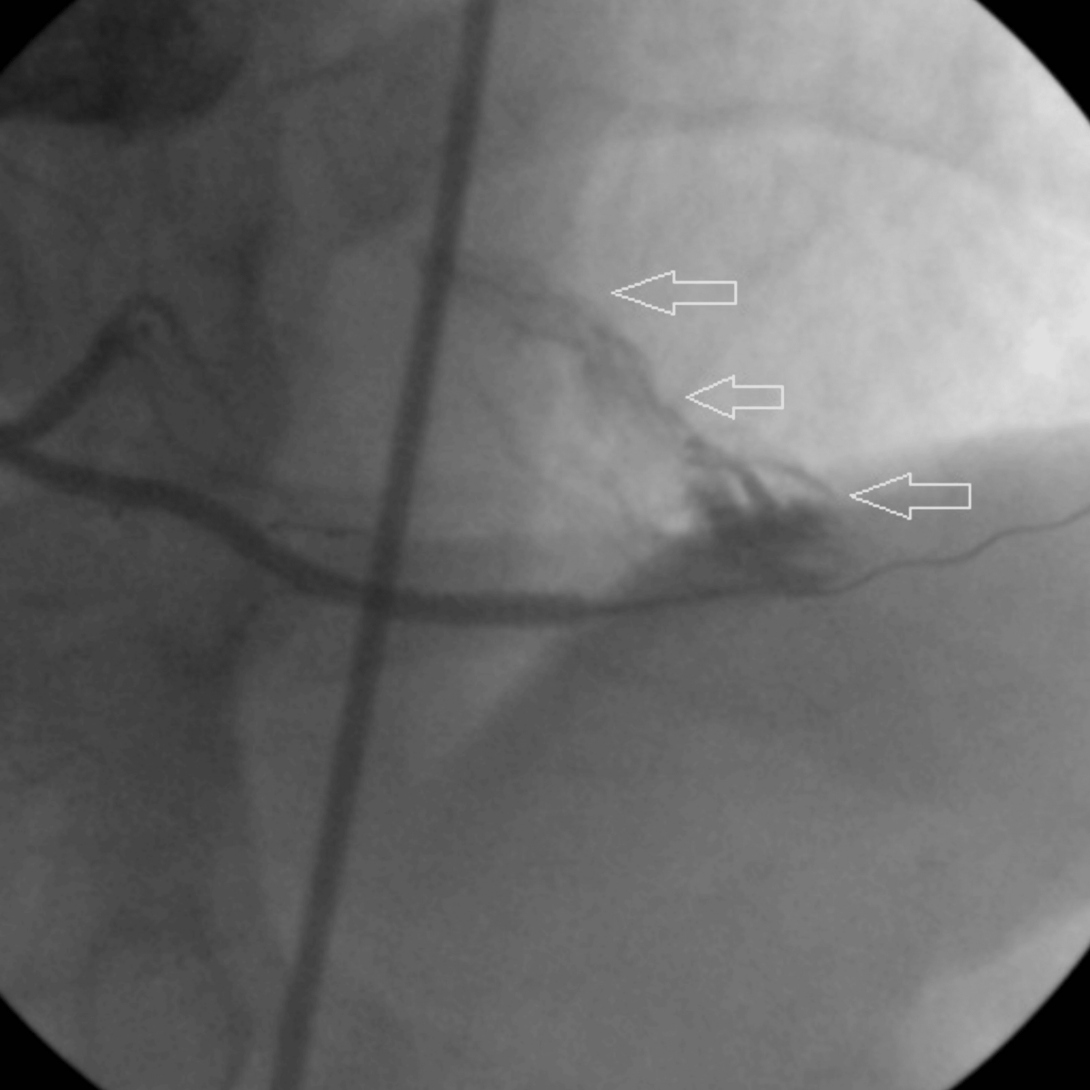
Arrows show increased fistula after prolonged ballooning.

The patient experienced an acute onset of chest pain and breathlessness. Repeated CAG revealed a significant fistula between the PDA and the left ventricle (Figure 7).

**Figure 8:**
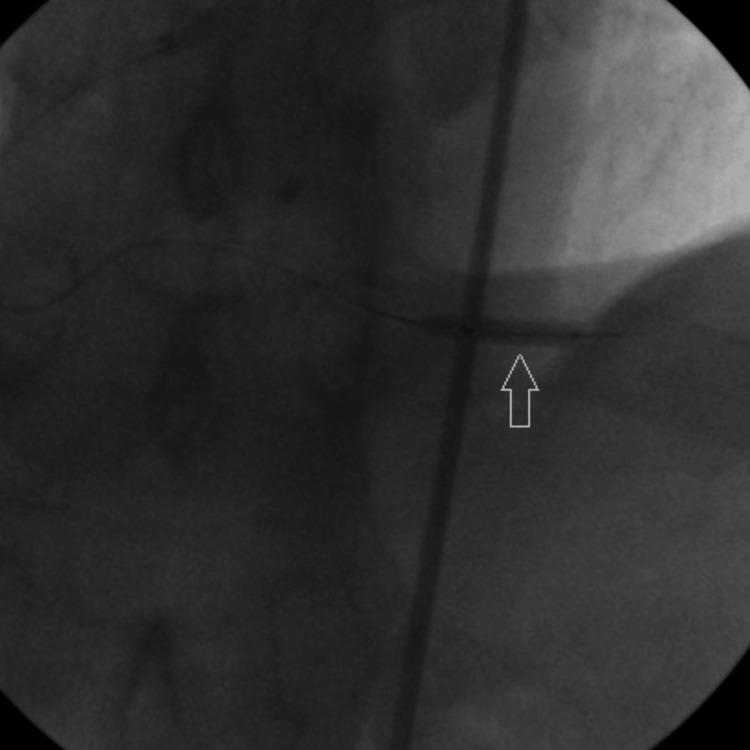
Arrow shows prolonged ballooning within formerly implanted stent.

Since his vital signs were stable and repeated echocardiography showed no pericardial effusion, we suspected a CAF between the PDA and the left ventricle, likely resulting from coronary artery perforation due to guidewire-induced trauma, subsequent stent implantation, and prolonged balloon inflation. Most likely, while navigating the occluded lesion, the guidewire inadvertently advanced deep into the left ventricle through either the septal branch or the distal segment of the PDA. The guidewire may have also entered a subintimal space. Subsequently, we attempted to locate the true lumen with a second floppy wire, but we were unable to do so. Given the significant leakage, we opted to close the CAF by inflating a balloon at the pre-perforation site. After slightly withdrawing the floppy wire, we occluded the CAF by performing extended balloon inflation for 5 minutes using a 3.0 mm × 15 mm balloon dilation catheter in the formerly implanted stent, just before the rupture zone (Figure 8).

**Figure 9:**
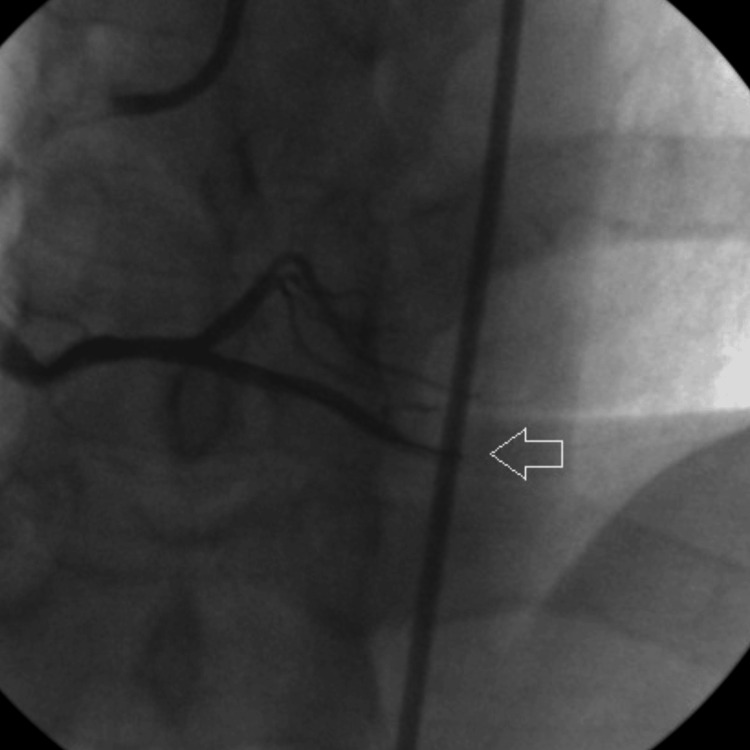
Arrow shows loss of distal flow of PDA. PDA: Posterior descending artery

Additionally, we lost the distal blood flow of the PDA (Figure 9).

The patient remained symptom-free during and after the fistula closure procedure. Follow-up echocardiography showed only hypokinesis of the baseline inferior wall, consistent with initial values. Two days later, the patient was discharged in good health without any complaints, continuing treatment with ASA, metoprolol, and statin. The patient has remained asymptomatic after 6 months of follow-up, with nearly normal echocardiographic results.

## Discussion

Incompatible wire tracking, wire-induced perforation, subintimal balloon inflation, and hyperextension of a coronary segment using a balloon or stent are probable causes of CAF [3-5,7,8]. In the patient, iatrogenic CAF between the PDA and the left ventricle occurred as a consequence of guidewire passage deep into the left ventricle through either the septal branch or the end of the short cruising distal segment of the PDA, followed by both stent implantation and balloon inflation. In the present case, we utilized a balloon and a drug-eluting stent suitable for the diameter of the coronary artery being treated, ensuring no over-dilation was performed. We employed a floppy guidewire to navigate the occluded lesion. While using stiff guidewires has been significantly associated with the risk of CAF, we believe that floppy guidewire was the primary contributing factor to the CAF observed in this case. Similarly, Banerjee and Patra reported a case involving a 57-year-old woman with critical two-vessel disease, where a floppy wire induced a CAF between the RCA and the right ventricle during PCI [7].

This experience indicates that we must exercise caution regarding such complications, even when using a floppy wire during PCI. The key point is what actions should be taken after CAF has occurred during PCI. Ellis types I and II perforations can be conservatively managed by reversing unfractionated heparin with protamine, prolonged balloon inflation, and utilizing coils and/or covered stents if necessary. Type III, IV, and V perforations may lead to significant complications, often necessitating more invasive interventions, such as emergency pericardiocentesis and cardiac surgery [2-9].

The case presented is unique as it exhibits features of type III, type IV, and likely type V perforations. In this situation, after extended ballooning at the rupture zone to close the fistula, we encountered a greater leak. We determined that the distal part of the floppy guidewire was not in the true lumen. Since we could not confirm the true lumen with a second wire despite several attempts, we opted not to perform the covered stent. After a swift discussion with the heart surgeon, we conducted prolonged balloon inflation within the previously implanted stent, just before the rupture zone, at the cost of losing distal blood flow to the PDA.

One might criticize this practice we undertook. However, the leakage was too significant to leave unaddressed. If we did not close it in some manner, we would face complications over time, including further myocardial ischemia, various cardiac arrhythmias, congestive heart failure, pulmonary artery hypertension, and endocarditis.

## Conclusions

This case highlights crucial information on how to manage a PCI-associated CAF in the context of ACS. Care must be taken in all instances, whether the lesion is simple or complex, concerning the development of CAF. The orientation and application of the materials utilized should be thoughtfully considered. The necessary precautions must be implemented promptly.
